# Involvement of a Velvet Protein FgVeA in the Regulation of Asexual Development, Lipid and Secondary Metabolisms and Virulence in *Fusarium graminearum*

**DOI:** 10.1371/journal.pone.0028291

**Published:** 2011-11-29

**Authors:** Jinhua Jiang, Xin Liu, Yanni Yin, Zhonghua Ma

**Affiliations:** Key Laboratory of Molecular Biology of Crop Pathogens and Insects, Institute of Biotechnology, Zhejiang University, Hangzhou, China; Seoul National University, Republic of Korea

## Abstract

The velvet protein, VeA, is involved in the regulation of diverse cellular processes. In this study, we explored functions of FgVeA in the wheat head blight pathogen, *Fusarium graminearum*,using a gene replacement strategy. The *FgVEA* deletion mutant exhibited a reduction in aerial hyphae formation, hydrophobicity, and deoxynivalenol (DON) biosynthesis. Deletion of *FgVEA* gene led to an increase in conidial production, but a delay in conidial germination. Pathogencity assays showed that the mutant was impaired in virulence on flowering wheat head. Sensitivity tests to various stresses exhibited that the *FgVEA* deletion mutant showed increased resistance to osmotic stress and cell wall-damaging agents, but increased sensitivity to iprodione and fludioxonil fungicides. Ultrastructural and histochemical analyses revealed that conidia of FgVeA deletion mutant contained an unusually high number of large lipid droplets, which is in agreement with the observation that the mutant accumulated a higher basal level of glycerol than the wild-type progenitor. Serial analysis of gene expression (SAGE) in the *FgVEA* mutant confirmed that FgVeA was involved in various cellular processes. Additionally, six proteins interacting with FgVeA were identified by yeast two hybrid assays in current study. These results indicate that FgVeA plays a critical role in a variety of cellular processes in *F. graminearum*.

## Introduction

*Fusarium graminearum* Schwabe [teleomorph *Gibberella zeae* (Schwein.) Petch], a homothallic ascomycete, is the major causal agent of Fusarium head blight (FHB), which is a devastating disease of cereal crops worldwide [1]. While yield loss caused by the disease is a major concern, the mycotoxins, such as deoxynivalenol (DON) and its derivatives, produced by the fungus in infected grains pose a serious threat to human and animal health [2]. Despite the high economic impact of FHB, efficient strategies for the management of FHB are not available yet, which could be explained in part by our limited information for *F. graminearum* biology. Therefore, a better understanding of regulation mechanisms of fungal development, virulence, and DON biosynthesis in *F. graminearum* will be essential to facilitate the development of efficient control strategies against FHB.

The velvet protein encoded by *VEA* gene has been shown to be involved in the regulation of diverse cellular processes, including control of asexual and sexual development as well as secondary metabolisms in several fungal species [3], [4]. The VeA was first characterized in *Aspergillus nidulans* as an inhibitor of light-dependent conidiation in 1960 [5], and was later shown to be a negative regulator of asexual development [6]. A *VEA* deletion mutant of *A. nidulans* failed to form fruiting bodies, and the opposite effect was observed when the gene was over-expressed, which confirmed that VeA is a positive regulator of sexual development and simultaneously a negative regulator of asexual development [7]. It is interesting that in *A. parasiticus*, genetically related to *A. nidulans*, deletion of *VEA* resulted to a reduction of conidiation [8]. These results indicate that the role of VeA in sexual development varies significantly among different fungal species.

In last few years, effects of VeA on secondary metabolism have been well investigated in *Aspergillus* spp. In *A. nidulans*, VeA is necessary for expression of the transcription factor *AflR*, which activates the mycotoxin sterigmatocystin biosynthesis gene cluster [9]. Similarly, in *A. parasiticus* and *A. flavus*, VeA is essential for the expression of two transcription factors *AflR* and *AflJ*, which are necessary for activation of aflatoxin biosynthesis genes. Consequently, the mycotoxin aflatoxin biosynthesis is completely blocked in *VEA* deletion mutants of these fungi [10], [11]. In addition to its multiple functions in secondary metabolism and fungal development, recent evidence showed that VeA negatively regulated catabolism of branched chain amino acid and ethanol metabolism at the transcriptional level [12].

VeA proteins are conserved throughout the fungal kingdom [13]. Recently, functions of VeA have been investigated in several other filamentous fungi including *Acremonium chrysogenum* [14], *Fusarium verticillioides* [13], [15], *Mycosphaerella graminicola* [16] and *Penicillium chrysogenum* [17]. In these fungal species, *VEA* deletion mutants present some new phenotypic characteristics, which were not described in *Aspergillus* spp. For example, deletion of *VEA* gene (*FvVE1*) in *F. verticillioides* suppressed aerial hyphal growth and reduced colony surface hydrophobicity on solid media. In addition, deletion of *FvVE1* markedly increased the ratio of macroconidia to microconidia [13]. The *VEA* deletion mutants of *M. graminicola* were hypersensitive to shaking [16]. In *F. fujikuroi*, FfVel1 (a homolog of VeA of *Aspergillus* spp.) can act as a positive regulator for biosyntheses of gibberellins, fumonisins and fusarin CA, simultaneously as a negative regulator for another secondary metabolite bikaverin [18]. Furthermore, *FfVel1* deletion mutants failed to infect rice seedlings [18]. In contrast, pathogenicity was not altered in the *VEA* deletion mutant of *M. graminicola* [16]. These studies indicate that functions of VeA in different fungal species may vary significantly.

The purpose of this study was to investigate functions of *FgVEA* gene encoding a VeA-homologous protein in *F. graminearum*. Although the role of VeA in controlling synthesis of secondary metabolites is a common feature, notably, in current study, we observed that the *FgVEA* deletion mutants of *F. graminearum* presented some new phenotypic characteristics, which were not previously described in other fungi.

## Results

### Sequence analysis of *FgVEA*

The *FgVEA* (*F. graminearum* genome accession number FGSG_11955.3) was originally identified through homology searches of the *F. graminearum* genome sequence by using BLASTP algorithm with the FvVe1 of *F. verticillioides* [13] as query. In *F. graminearum* genome database, FGSG_11955.3 missed a 225-bp fragment. After sequenced the full length genomic DNA and cDNA sequence for *FgVEA*, we found that the gene including one intron is 1,656-bp in length, and encodes a 532-amino-acid protein. The predicted amino acid sequence of FgVeA shares 79%, 78%, 52%, and 47% identities to FfVel1 of *F. fujikuroi*, FvVe1 of *F. verticillioides*, VeA of *A. nidulans*, and PcVelA of *P. chrysogenum*, respectively. Alignment of predicted amino acid sequences showed that N-terminal regions of VeA from different fungi including *F. graminearum* are highly conserved (Fig. S1B). Further *in silico* analyses demonstrated that FgVeA has a putative pat4 nuclear localization signal (NLS) from amino acids 474 to 477.

### Deletion of FgVEA in F. graminearum

**Figure 1:**
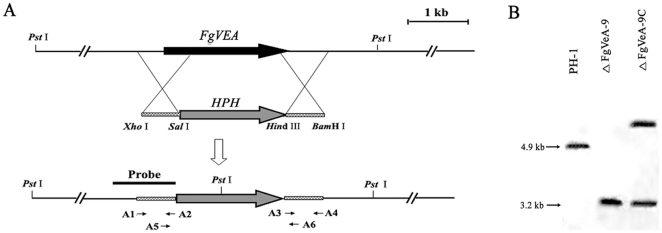
Schematic representation of the *FgVEA* deletion strategy. (A) *FgVEA* and hygromycin resistance cassette (*HPH*) are denoted by large black and gray arrows, respectively. Annealing sites of PCR primers are indicated with arrows (see supplementary Table 1 for the primer sequences). **(B)** A 1,162-bp fragment of *FgVEA* was used as a probe in Southern blot hybridization analysis. Genomic DNA preparations of the wild-type PH-1, the *FgVEA* deletion mutant ΔFgVeA-9, and the complement strain ΔFgVeA-9C were digested with *Pst* I.

To investigate functions of the velvet protein FgVeA in *F. graminearum*, we generated gene deletion mutants using a homology recombination strategy (Fig. 1A). Among thirteen hygromycin-resistant transformants, eight *FgVEA* deletion mutants were identified by PCR analysis with the primer pair A5 + A6 (Table S1). The primer pair amplified 1,759- and 1,537-bp fragments from *FgVEA* deletion mutants and the wild-type progenitor PH-1, respectively. When probed with a 1,162-bp DNA fragment of *FgVEA*, the deletion mutant ΔFgVeA-9 had an anticipated 3,213-bp band, but lacked a 4,912-bp band which was present in the progenitor (Fig. 1B). The Southern hybridization pattern confirmed that the ΔFgVeA-9 is a null mutant resulting from a single homologous recombination event at the *FgVEA* locus. The complemented strain ΔFgVeA-9C contains a single copy of wild-type *FgVEA*, which was inserted into genome of the *FgVEA* deletion mutant ΔFgVeA-9 (Fig. 1B).

### Effects of FgVeA on hyphal growth and pigment formation in *F. graminearum*

**Figure 2:**
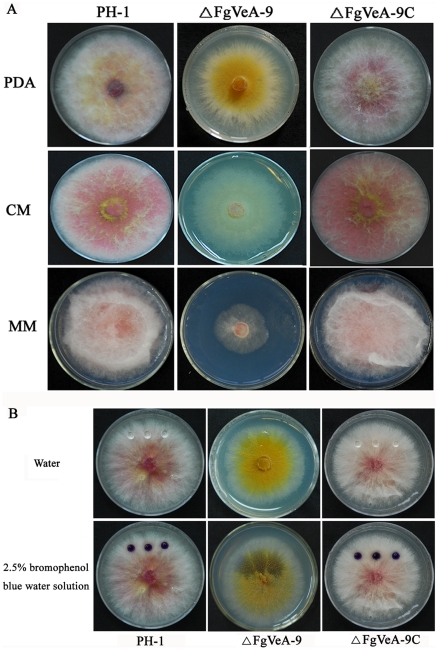
Impact of *FgVEA* on colony morphology and pigment formation. **(A)** The wild-type strain PH-1, *FgVEA* deletion mutant ΔFgVeA-9, and complemented strain ΔFgVeA-9C were grown on solid media, PDA, CM, MM for 4 days at 25°C. (**B)** On each of the fungal colonies, 20 µl of water or 2.5% bromophenol blue solution was pipetted on the colony surface and photographed 10 min later. Spherical water droplets formed on colonies of PH-1 and ΔFgVeA-9C, whereas the droplet dispersed immediately on colonies of ΔFgVeA-9.

The deletion of *FgVEA* dramatically affected colony morphology of *F. graminearum* on solid media. The mycelial growth rate of ΔFgVeA-9 was significantly slower than that of wild type progenitor PH-1 and the complemented strain ΔFgVeA-9C on MM medium (Fig. 2A). In addition, the ΔFgVeA-9 exhibited reduced aerial hyphal growth on solid media PDA, CM and MM (Fig. 2A), although scanning electron microscopy examination showed that the hyphae of ΔFgVeA-9 were not significantly different from those of wild type progenitor (data not shown). The phenotypic defects of ΔFgVeA-9 mutant on solid media were restored by genetic complementation with the wild-type *FgVEA* in the complemented strain ΔFgVeA-9C (Fig. 2A).

The hydrophobic property on the cell surface is a distinguishable feature of aerial hyphae and contributes to hyphal formation in many fungal species [19], [20]. Deletion of *FgVEA* led to inhibition of aerial hyphae gorwth, which suggested a reduction of hydrophobicity on cell surface of the mutant. To confirm this deduction, 20 µl of water was placed on the colony surface of each strain grown on the solid medium PDA. As shown in Fig. 2B, the water formed spherical droplets on the colony of wild-type progenitor without extending or being absorbed for at least 30 min. In contrast, the water was absorbed into the mycelia of ΔFgVeA-9 within 10 sec. The absorption difference was easily visualized when 2.5% bromophenol blue water solution was placed on the colony surface (Fig. 2B). These results indicate that FgVeA is important for colony surface properties in *F. graminearum.*

**Figure 3:**
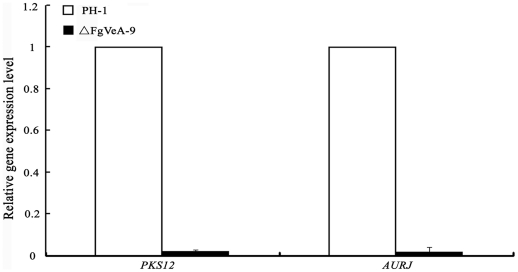
Relative expression levels of *PKS12* and *AURJ* in the *FgVEA* deletion mutant ΔFgVeA-9. RNA samples were extracted from mycelia of each strain after grown in potato dextrose broth for 2 days. The relative expression of *PKS12* and *AURJ* in ΔFgVeA-9 is the relative amount of cDNA of each gene in the wild-type strain. Line bars in each column denote standard errors of three experiments.

On CM medium, it was clear that ΔFgVeA-9 revealed a significant reduction in red pigment formation (Fig. 2A). To further confirm this observation, we assayed the expression of *PKS12* and *AURJ* genes encoding a type I polyketide synthase and O-methyltransferase, respectively, which are necessary for red pigment biosynthesis [21]. Quantitative real-time PCR (qRT-PCR) analyses showed that expression levels of both *PKS12* and *AURJ* in ΔFgVeA-9 were decreased by 98% as compared to those in wild type progenitor PH-1 (Fig. 3). These results indicate that FgVeA was involved in the regulation of pigment biosynthesis in *F. graminearum*.

### Effects of FgVeA on conidial differentiation and germination

**Figure 4:**
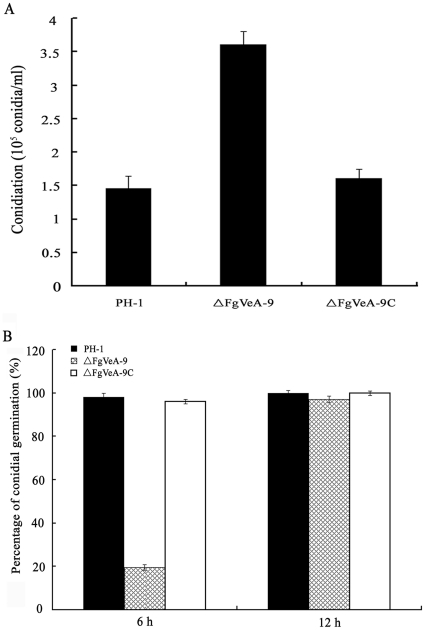
Impact of *FgVEA* on conidiation and conidial germination of *F. graminearum*. (A) Conidia were quantified after incubation of the wild-type strain PH-1, *FgVEA* deletion mutant ΔFgVeA-9, and complemented strain ΔFgVeA-9C in 10 ml mung bean liquid medium for 4 days in a shaker. **(B)** Percentages of germinated conidia of PH-1, ΔFgVeA-9, and ΔFgVeA-9C after incubated in 2% glucose for 6 h or 12 h. Bars denote standard errors from three repeated experiments.

**Figure 5:**
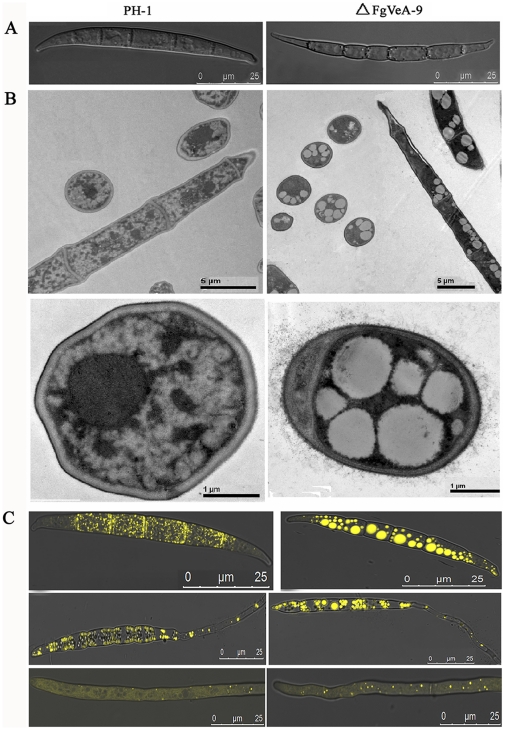
Ultrastructural and histochemical analyses of lipid droplets within conidia and hyphae of the mutant ΔFgVeA-9. (**A)** Differential interference contrast (DIC) images of conidia were captured with an electronic microscope. **(B)** Lipid drops within conidia of the wild type PH-1 and the ΔFgVeA-9 mutant examined with a transmission electronic microscope. **(C)** Lipid drops in conidia (top), germinating conidia (middle), and hyphae (bottom) were stained with Nile Red and examined under a microscope with episcopic fluorescence.

The VeA had been found to regulate asexual development in several other fungal species [4]. Consequently, we examined the conidiation, conidial germination, and cellar structure for the *FgVEA* deletion mutant ΔFgVeA-9. In MBL medium, ΔFgVeA-9 produced significantly more conidia than the wild type progenitor or the complemented strain (Fig. 4A). When cultured the conidia in 2% glucose, only approximately 20% conidia of ΔFgVeA-9 were able to germinate within 6 hr of incubation, but almost all conidia of wild type progenitor or the complemented strain germinated under the same condition. When incubation time was extended to 12 h, all the conidia of ΔFgVeA-9 were able to germinate (Fig. 4B), and form normal unbranched germ tubes, indicating that deletion of *FgVEA* led to a delay in conidial germination. Scanning electron microscopic examination showed that conidia of ΔFgVeA-9 were slightly slender than those of wild type PH-1 (Fig. 5A). In order to characterize the conidia of ΔFgVeA-9 in detail, we examined conidial structure using transmission electron microscopy. As shown in Fig. 5B, a few large lipid droplets were observed in ΔFgVeA-9 conidia, but not in those of wild type strain. The lipid droplets were further verified by histochemical staining with Nile Red. The large discrete lipid droplets were highlighted in the ungerminated and germinating conidia of ΔFgVeA-9, but not in the wild-type strain (Fig. 5C). The large discrete lipid droplets were degraded in the hyphae of ΔFgVeA-9 (Fig. 5C). In the following serial analysis of gene expression (SAGE) experiment, we also paid attention on the expression of the genes involved in fatty acid biosynthesis and metabolism. As shown in Table S2, among 41 genes, 25 and 1 genes involved in fatty acid metabolism were up- and down-regulated, respectively, more than five folds. These results strongly indicate that FgVeA was involved in the regulation of lipid metabolism in *F. graminearum*.

### Sensitivity of *FgVEA* deletion mutant to osmotic stresses and fungicides

**Figure 6:**
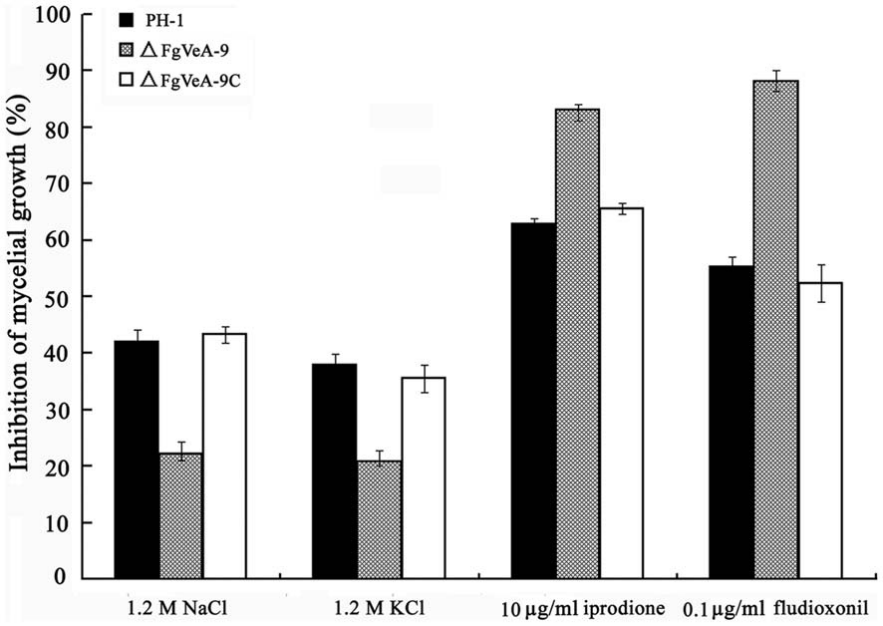
Sensitivity of the PH-1, ΔFgVeA-9 and ΔFgVeA-9C to osmotic stresses and fungicides. Osmotic stresses were mediated by addition of 1.2 M NaCl or 1.2 M KCl in potato dextrose agar (PDA) medium. The fungicides iprodione and fludioxonil were added into PDA at 10 µg/ml and 0.1 µg/ml, respectively. Bars denote standard errors from three repeated experiments.

In *F. verticillioides*, the *FvVE1* deletion mutants exhibited a dramatic increase in production of aerial hyphae and radial growth on solid media amended with osmotic stabilizers NaCl, KCl, sorbitol, and sucrose [13]. Thus, we examined sensitivity of ΔFgVeA-9 to osmotic stresses mediated by these osmotic stabilizers. As shown in Fig. 6, ΔFgVeA-9 exhibited significantly increased resistance to ionic osmotic stress mediated by 1.2 M NaCl and 1.2 M KCl. Previous studies have shown that phenylpyrrole and dicarboximide fungicides activate the high osmolarity glycerol (HOG) pathway [22], [23], and the fungicide-resistant mutants of *F. graminearum* revealed increased sensitivity to osmotic stabilizers, while the fungicide-sensitive strain was tolerant to osmotic stress [24]. Therefore, we were interested in determining the sensitivity of ΔFgVeA-9 to the phenylpyrrole fungicide fludioxonil and the dicarboximide fungicide iprodione. As expected, ΔFgVeA-9 showed increased sensitivity to the fludioxonil and iprodione (Fig. 6).

**Figure 7:**
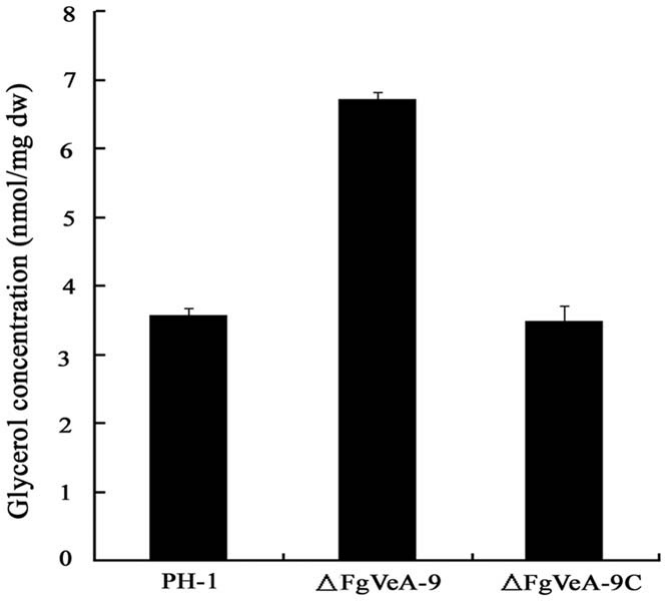
Effects of FgVeA on the glycerol biosynthesis. Intracellular glycerol concentration (nmol/mg dried mycelia) in mycelia of the wild-type strain PH-1, *FgVEA* deletion mutant ΔFgVeA-9, and complemented strain ΔFgVeA-9C were analyzed after incubation in PDB for 2 days. Bars denote standard errors from three repeated experiments.

It has been reported that osmotic stress can induce glycerol accumulation in fungi via the HOG-like pathway [25]. We therefore analyzed glycerol accumulation in mycelia of the mutant ΔFgVeA-9. As shown in Fig. 7, the level of glycerol concentration in ΔFgVeA-9 was significantly higher than that in the wild-type PH-1 and the complemented strain ΔFgVeA-9C, which could partially explain the reason why ΔFgVeA-9 exhibited increased resistance to osmotic stresses.

### Resistance of the *FgVEA* deletion mutant to cell wall damaging agents

**Figure 8:**
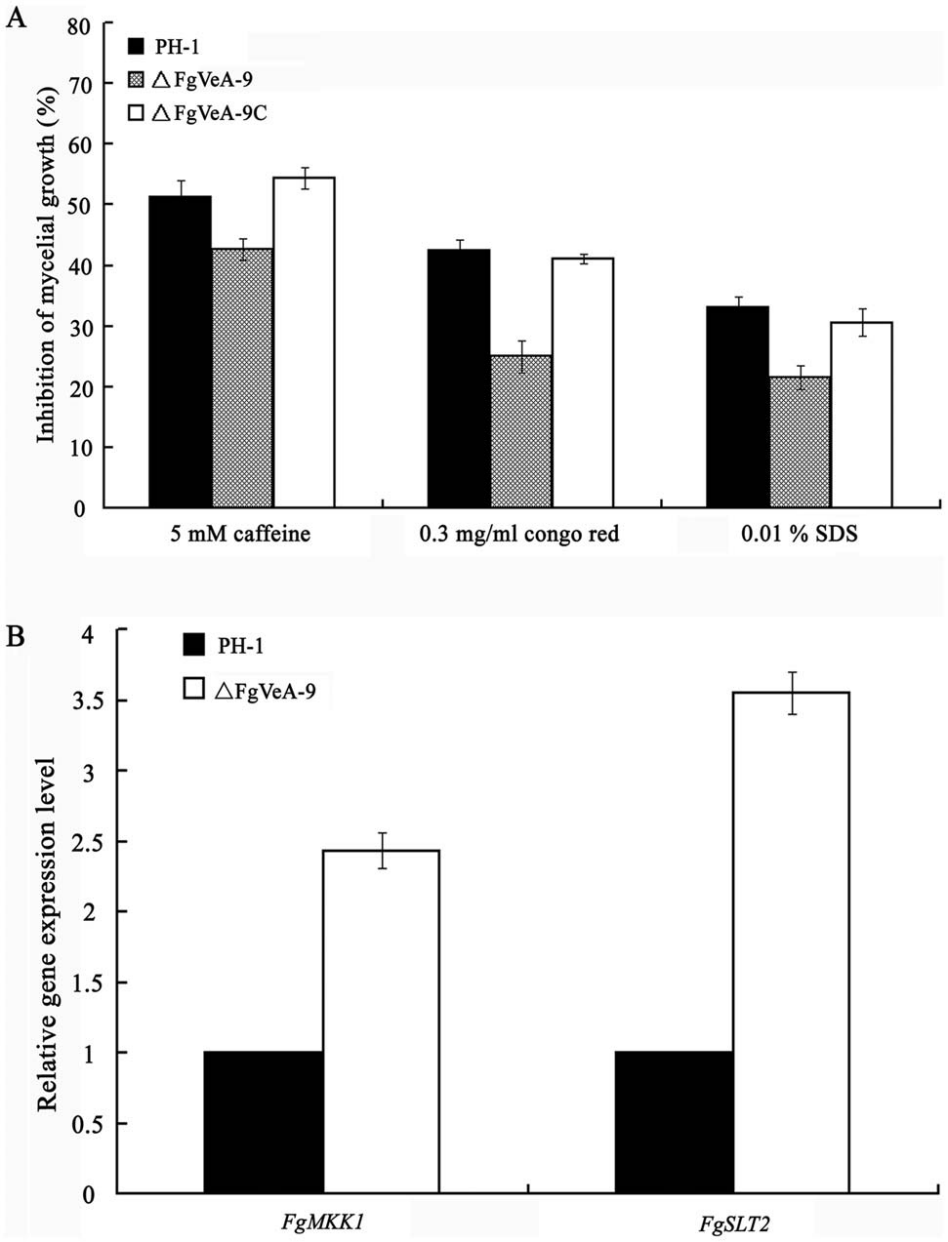
Effects of FgVeA on cell wall integrity of *F. graminearum*. **(A)** Sensitivity of the wild type PH-1, *FgVEA* deletion mutant ΔFgVeA-9, and complemented strain ΔFgVeA-9C to cell wall damaging agents. **(B)** Relative expression levels of *FgSLT1* and *FgMKK1* in PH-1 and mutant ΔFgVeA-9. The relative expression of *FgSLT1* and *FgMKK1* in ΔFgVeA-9 is the relative amount of cDNA of each gene in the wild-type strain. Line bars in each column of each figure denote standard errors of three repeated experiments.

The deletion of *FgVEA* led to an increase in resistance to osmotic stabilizers, which suggests that FgVeA might be involved in the regulation of cell member and/or cell wall integrity. To address this, we determined sensitivity of ΔFgVeA-9 to cell member damaging agent SDS, and to cell wall damaging agents: congo red and caffeine. Compared to the wild type progenitor and the complemented strain, ΔFgVeA-9 displayed increased resistance to these compounds (Fig. 8A). To further confirm the involvement of FgVeA in the regulation of cell wall integrity (CWI) pathway, we determined the expressions of *FgMKK1* (FGSG_07295) and *FgSLT2 *(FGSG_10313), which are homologous to the *S. cerevisiae* CWI core element genes, *Mkk1* and *Slt2*, respectively. As shown in Fig. 8B, expression levels of *FgMKK1* and *FgSLT2* in ΔFgVeA-9 were 2.43 and 3.55-folds, respectively, higher than those in the wild-type strain. These results further supported that FgVeA was associated with the CWI pathway.

### Role of FgVeA in the regulation of deoxynivalenol (DON) biosynthesis

**Figure 9:**
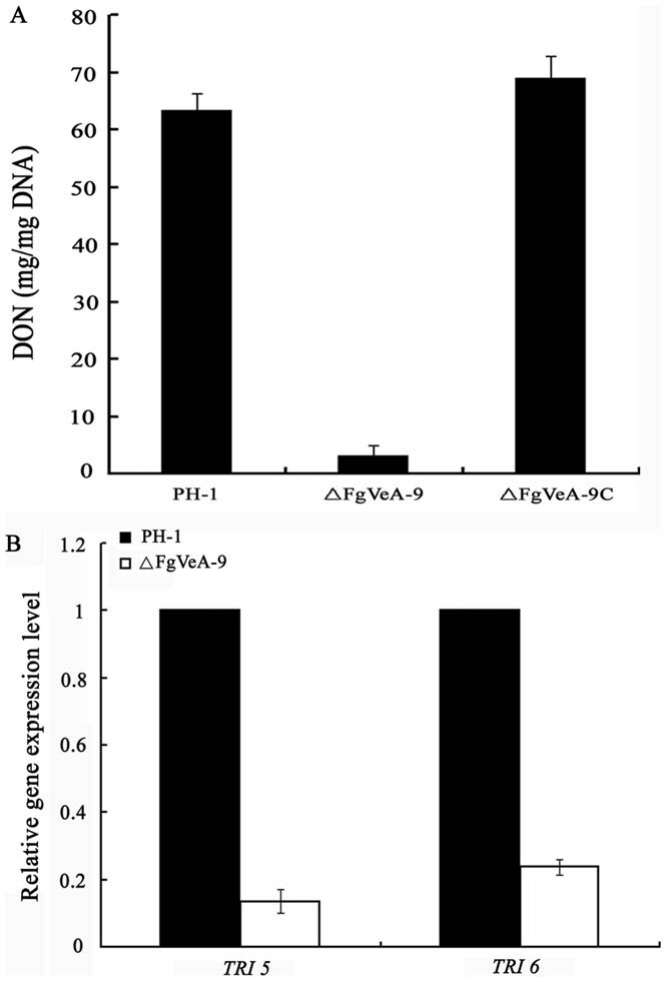
Effects of FgVeA on the biosynthesis of DON. (A) Amount of DON (per mg fungal DNA) produced by *FgVEA* deletion mutant ΔFgVeA-9, and complemented strain ΔFgVeA-9C was detected in infected wheat kernels after 20 days of incubation. Line bars in each column denote standard errors of three repeated experiments. **(B)** Relative expression levels of *TIR5* and *TRI6* in PH-1 and ΔFgVeA-9. The relative expression of *TIR5* or *TRI6* in ΔFgVeA-9 is the relative amount of cDNA of each gene in the wild-type progenitor. Line bars in each column denote standard errors of three repeated experiments.

Previous studies have shown that VeA proteins were involved in the regulation of secondary metabolism in several fungi [4]. *F. graminearum* produces various secondary metabolites including the mycotoxin DON. Therefore, we analyzed DON biosynthesis in ΔFgVeA-9. When cultured on wheat kernels for 20 days, the amount of DON produced by the wild-type strain were 21 times higher than that produced by ΔFgVeA-9 (Fig. 9A). Complementation of the gene restored the ability of the fungus to produce DON production. To further confirm this finding, we assayed the expression of *TRI5* and *TRI6* by quantitative real-time PCR (qRT-PCR) using RNA samples isolated from mycelia grown in GYEP medium. The expression levels of *TRI5* and *TRI6* in the mutant ΔFgVeA-9 was decreased by 87% and 76%, respectively, as compared to those in wild type progenitor (Fig. 9B). These results indicate that FgVeA was necessary for the regulation of DON biosynthesis in *F. graminearum*.

### FgVeA is essential for virulence of *F. graminearum*

**Figure 10:**
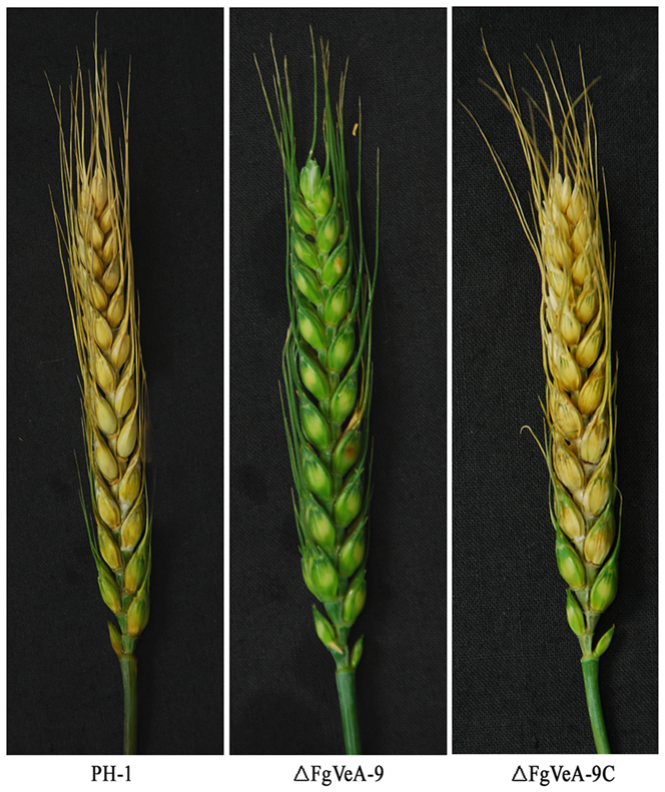
Virulence of the wild type PH-1, *FgVEA* deletion mutant ΔFgVeA-9, complemented strain ΔFgVeA-9C on flowering wheat heads. Wheat heads were point-inoculated with conidial suspension of each strain, and infected wheat heads were examined 15 days after inoculation.

DON has been identified as a virulence factor in *F. graminearum* [26]–[28]. Since the *FgVEA* deletion mutants were impaired in DON biosynthesis, we further analyzed the virulence of *FgVEA* deletion mutant by point inoculating conidial suspension on flowering wheat head. Fifteen days after inoculation, ΔFgVeA-9 caused infection only in the inoculated spikelet, but not in nearby spikelets (Fig 10). Under the same conditions, however, scab symptoms developed in more than 90% spikelets when wheat heads were point-inoculated with the wild-type PH-1, or the *FgVEA* complemented strain FgVeA-9C (Fig. 10).

### Serial analysis of gene expression (SAGE) reveals that FgVeA is associated with various metabolism pathways

**Figure 11:**
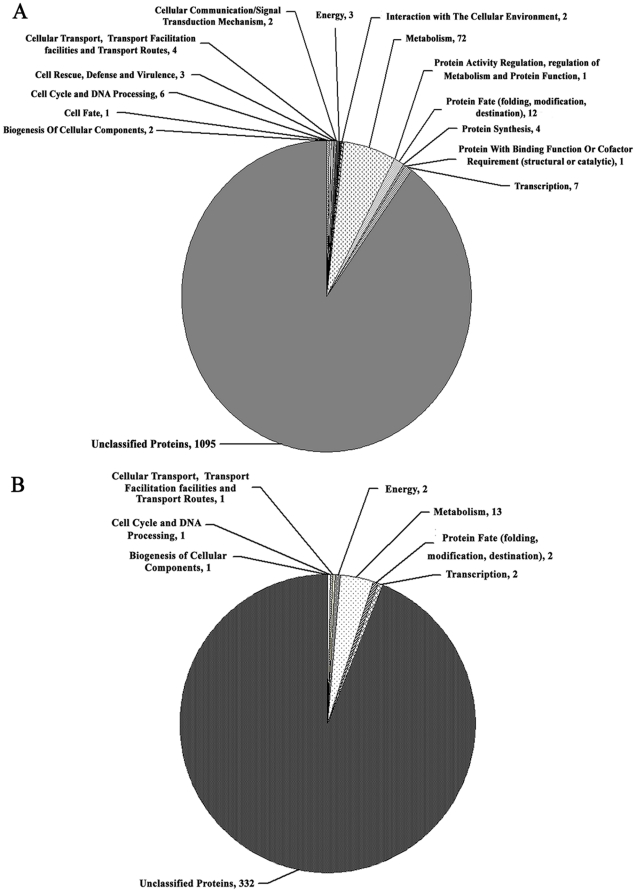
Pie chart grouping the genes up- and down-regulated in expression in ΔFgVeA compared with PH-1. (A) A total of 1,215 genes were up-regulated more than 5 folds in the mutant ΔFgVeA-9 compared with wild-type PH-1. (B) A total of 354 genes were down-regulated more than 5 folds in the mutant ΔFgVeA-9 compared with wild-type PH-1. The expressions of genes were detected by the serial analysis of gene expression method.

To further elucidate the function of FgVeA as well as identify genes that it may impact, we conducted SAGE assays for ΔFgVeA-9 and the wild-type progenitor PH-1. After removal of low quality (<3) tags, a total of 125,406 and 131,640 distinct tags were obtained for PH-1 and ΔFgVeA-9, respectively. Among these distinct tags, 70.02% and 75.5% can be uniquely mapped to the reference sequences for PH-1 and ΔFgVeA-9, respectively. For SAGE data, the analysis is usually limited to a predefined tag showing at least 5-fold difference in abundance at a *P* value ≤0.05 [29]. With this criterion, we identified 1,215 genes up-regulated (>5-folds) and 354 genes down-regulated (<0.2-folds) in ΔFgVeA-9 compared to PH-1. To obtain better understanding of the overall gene expression profile, the up- and down-regulated genes were mapped in the chromosomes, and they seemed to be distributed evenly in the chromosomes (Fig. S2). Using the FunCat program (http://mips.helmholtz-muenchen.de/proj/funcatDB/search_main_frame.html), the up- and down-regulated genes were further grouped into several functional categories, and most genes were classed as “unknown function”. Among the 120 classified genes, which were up-regulated in ΔFgVeA-9, 72 (60%) were grouped into the functional category of metabolism (Fig. 11A). Among the 22 classified down-regulated genes, again, 13 (59%) were associated with various metabolisms (Fig. 11B). In addition, expressions of 26 genes associated peroxisome biogenesis and SNARE interactions in vesicular transport pathway were changed dramatically in the *FgVEA* mutant (Figs. S3A and B). These results further support that FgVeA is involved in various cellular processes.

### FgVeA interact with several proteins containing methyltransferase domain

**Figure 12:**
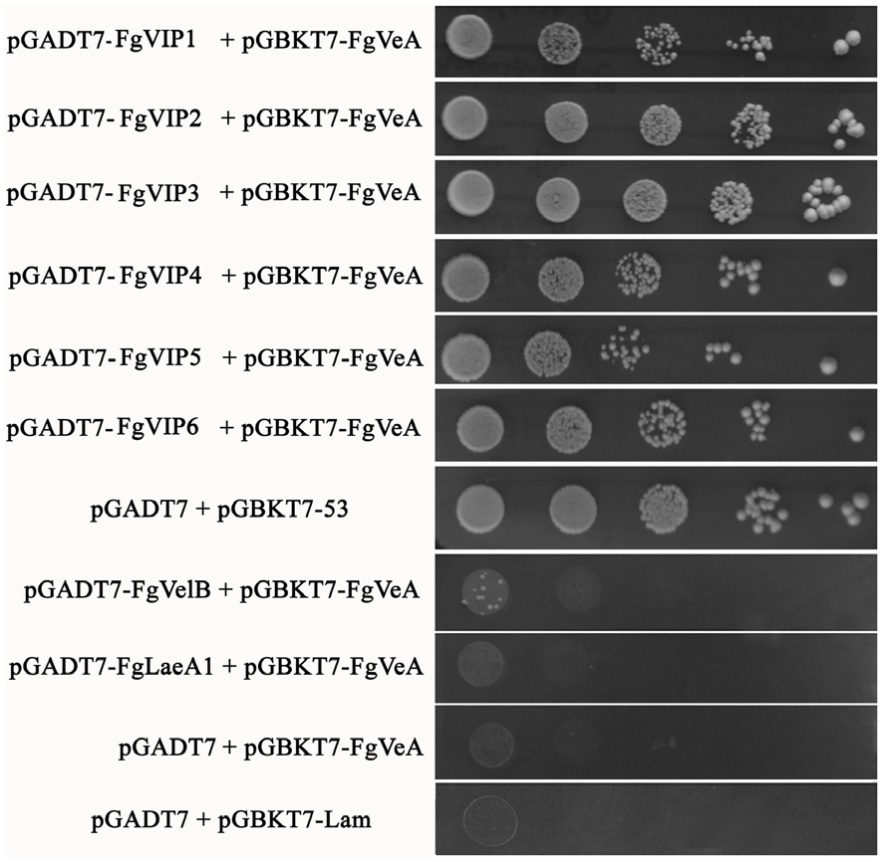
Yeast two-hybrid analysis of the interaction between FgVeA, FgVelB and FgLaeA1 or six FgVeA interacting proteins (named FgVIP1-6). The pair of plasmids pGBKT7-53 and pGADT7 was served as a positive control. The pairs of plasmids pGBKT7-Lam and pGADT7, pGADT7 and pGBKT7-FgVeA were used as negative controls. Growth of the transformed yeast was assayed on the medium containing 5 mM 3-aminotriazole (3-AT), but lacking His, Leu and Trp. Columns in each panel represent serial decimal dilution.

Recent work in *A. nidulans* indicated that the positive regulation of secondary metabolites is most likely achieved through the physical interactions of VeA with the velvet-like protein VelB and the putative methyltransferase LaeA in nucleus [30]. Yeast two-hybrid analysis confirmed the VeA-VelB and VeA-LaeA interactions, where VelB and LaeA do not interact in *A. nidulans*, suggesting that VeA acts as a bridge between VelB and LaeA [30]. Genome-wide search for the homolog of VelB and LaeA in *F. graminearum* showed that the fungus contains a VelB homolog (here named *FgVelB*, FGSG_01362), and a LaeA homolog designed *FgLaeA1* (FGSG_00657). Deletion of *FgLaeA1* led to a reduction in red pigment formation on PDA medium (Fig. S4). Surprisingly, yeast two-hybrid assay showed that FgVeA did not interact with FgVelB or FgLaeA1 (Fig. 12). Using yeast two-hybrid approach, we did identify six FgVeA interacting proteins (designed FgVIPs), that are homologous to FgLaeA1 (Fig. 12). All these six FgVIP proteins [FgVIP1 (FGSG_07660), FgVIP2 (FGSG_03525), FgVIP3 (FGSG_05685), FgVIP4 (FGSG_03567), FgVIP5 (FGSG_08741), and FgVIP6 (FGSG_03011)] contain a conserved methyltransferase domain. It was surprised that except for a slight change in pigment formation, the deletion mutants of these six genes were indistinguishable from the wild type progenitor on PDA plates (Fig. S4), and all mutants were pathogenic on wheat head (data not show). These results strongly indicate that the velvet complex in *F. graminearum* was quite different from that in *A. nidulans*. An important challenge at this time is to elucidate the dynamics and functions of this large protein complex in *F. graminearum*.

## Discussion

Recent studies in several filamentous fungi have shown that the VeA protein plays an important role in fungal growth, colony morphology, development, and secondary metabolism. However, certain variations in the role have been observed in different fungi even within the genus of *Aspergillus* [4]. For example, complementation of the *A. nidulans VEA* deletion mutant with the velvet gene from *F. verticillioides* could not rescue the wild-type phenotypes of *A. nidulans*, indicating species-specific functions of VeA in different fungi [4]. Bioinformatic analyses have demonstrated that fungal VeA proteins consist of conserved N-terminal and variable C-terminal regions, which may be responsible for conserved and species-specific functions of VeA, respectively. Consistent with conserved N-terminal as well as variable C-terminal functions of the velvet proteins, in this study, we found some of the phenotypes in *FgVEA* mutant were similar to those reported in other fungi, while others were novel and unique to *F. graminearum*.

The first study of VeA ortholog in other fungal species beyond the genus *Aspergillus* was conducted by Li *et al.* for the plant pathogen *F. verticillioides* [13]. In *F. verticillioides*, conidia of *FvVE1* deletion mutant exhibited three unnormal types of germination: hyperbranched hyphae, microcycle conidiation and yeast-like growth [13]. Different from *F. verticillioides*, in current study, we found that deletion of *F. graminearum FgVEA* led to a delay in conidial germination, but conidia of *FgVEA* deletion mutant germinated to form normal unbranched germ tubes. In *F. verticillioides*, deletion of *FvVE1* caused a notable activation of conidiation and increased the ratio of macroconida to microconidia [13]. In this study, we also observed that the *FgVEA* deletion mutant of *F. graminearum* produced significant more conidia than wild type progenitor. In contrast, deletion of *FfVel1* in *F. fujikuroi* led to a significant reduction in conidiation [18]. The opposite effect of VeA on conidiation had also been found in *Aspergillus* species. In *A. nidulans* and *A. flavus*, deletion of *VEA* resulted in an increase in asexual development [6], [31]. However, conidiation was reduced when *VEA* was deleted in *A. parasiticus*, a species genetically related to *A. flavus* [8]. These results strongly indicated that the roles of VeA in conidiation and conidial germination vary significantly in different fungal species.

A previous study of *F. verticillioides* showed that deletion of the *FvVE1* gene resulted in decreased hydrophobicity of the colony surface and impairment in aerial hyphae formation [13]. Similarly, we observed a significant reduction of aerial hyphal growth and hydrophobicity in *FgVEA* mutant compared to the wild-type progenitor PH-1. The reduced hydrophobicity on cell surface in *FgVEA* deletion mutant suggested an alteration in cell wall composition. We therefore tested sensitivity of the *FgVEA* deletion mutant to cell wall damaging agents, congo red and caffeine. As expectedly, the *FgVEA* deletion mutant showed resistant to cellulose-binging chemical congo red and to caffeine, which is in agreement with the overexpression of 1,3-beta-glucan synthase gene, *FgGLS2* (FGSG_07946) in the mutant (Table S2). These results indicated that FgVeA had an important role in maintaining normal cell wall composition and integrity.

Secondary metabolism had been reported to be positively regulated by velvet proteins in several fungi. *F. graminearum*, a pathogen of important cereal crops, produces various secondary metabolites including the trichothecene mycotoxin, DON [32]. In this study, we observed that FgVeA positively regulated expression of DON biosynthesis genes and DON production. Similar to DON production, the *FvVE1* deletion mutant of *F. verticillioides* produced significantly less gibberellin than the wild type strain did [18]. The dramatic down regulation of secondary metabolites in *Fusarium* spp. was consistent with the observation from *Aspergillus* spp., where VeA had been found to activate production of mycotoxin sterigmatosystin in *A. nidulans* [33], aflatoxin and cyclopiazonic acid in *A. flavus* [31], [34] as well as aflatoxin in *A. parasiticus* [8].

A previous study showed that the *FvVE1* deletion mutant of *F. verticillioides* produced more red pigment (polyketide bikaverin) than the wild type strain. In contrast, the *FvVE1* overexpression mutant showed significantly less coloration. Consistently with these observations, expressions of bikaverin cluster genes were significantly up-regulated in the *FvVEl* deletion mutant and down-regulated in the overexpression mutant. *F. graminearum* also produced a red pigment, the polyketide aurofusarin [35]. In contrast to the bikaverin biosynthesis in *F. verticillioides*, in current study, we found that the *FgVEA* deletion mutant produced dramatically less red pigment on solid media. Furthermore, expressions of seven aurofusarin biosynthesis genes were down-regulated significantly in the mutant (Table S2). These results indicate that the role of VeA in the regulation of pigment biosynthesis is species-dependent.

In response to osmotic pressure, fungi usually accumulated glycerol within their cells via HOG pathway to increase the internal turgor pressure [36]. In current study, we found that the *FgVEA* deletion showed increased tolerance to osmotic stress mediated by NaCl and KCl. This finding was in agreement with the observation that the mutant accumulated a higher level of glycerol than the wild-type progenitor. These results suggest that FgVeA was associated with the HOG pathway in *F. graminearum*.

In this study, phenotypic characterization of the *FgVEA* deletion mutant showed that FgVeA was essential for virulence of *F. graminearum*. The impairment in virulence of *FgVEA* deletion mutant appeared to be due to defects in multiple regulatory functions. First, the deletion of *FgVEA* led to a delay in conidial germination and retardation in mycelium growth. Second, the *FgVEA* mutant produced a dramatically low level of DON, which played a significant role in spread of the fungus within a spike [37]. In addition, it had been demonstrated that the hydrophobic property on the cell surface and normal fungal cell wall were important for viability, fungal morphology, and virulence [19], [38], the reduced hydrophobicity of cell surface in *FgVEA* deletion mutant may partially lead to impairment in virulence of the mutant on wheat head. The involvement of VeA protein in virulence had also been reported in *F. fujikuroi* [18], but not in *M. graminicola*. The F*fVel1* mutant of *F. fujikuroi* was nonpathogenic on rice seedlings; but in *M. graminicola*, the *VEA* deletion mutant remained high virulence on wheat leaves [16]. These results indicated that the roles of VeA in pathogenicity vary significantly in different fungi.

In this study, we observed that conidia of ΔFgVeA contained more lipid droplets than the wild-type progenitor. SAGE data also showed that an increased number of genes associated with fatty acid biosynthesis and metabolism were up-regulated significantly in the mutant (Table S2). To our knowledge, it was the first report that VeA was involved in lipid metabolism in fungi. Interestingly, previous analysis in our laboratory revealed that the deletion of a type 2C protein phosphatase gene *FgPTC3* also led to accumulation of large lipid droplets in *F. graminearum* conidia [39]. Furthermore, several phenotypes of the *FgVEA* deletion mutant, including reduction in aerial hyphal formation and DON biosynthesis, resistance to cell wall damaging agents, and impairment in pathogenicity, were similar to those of the *FgPTC3* deletion mutant. These results indicated that FgPtc3 and FgVeA had some common functions in regulation of various cellular processes. This inference was further supported by SAGE analyses of gene expression profiling in *FgPTC3* and *FgVEA* mutants. Among 1215 up-regulated genes in the *FgVEA* deletion mutant, 721 genes (59.3%) were also up-regulated in the *FgPTC3* deletion mutant. Again 354 down-regulated genes in the *FgVEA* deletion mutant, 218 genes (61.6%) were also down-regulated in the *FgPTC3* deletion mutant (Fig. S5). The significant overlap in genes regulated by FgVeA and FgPtc3 strongly indicated that both components were involved in some common signaling pathways in *F. graminearum*. Thus, it will be interesting to investigate relationships between these two components and their downstream network in *F. graminearum*, which would be helpful in understanding biology of *F. graminearum.*

## Materials and Methods

### Strains and culture conditions

*F. graminearum* wild-type strain PH-1 was used as a parental strain for transformation experiments. The wild-type strain and transformants generated in this study were grown on potato dextrose agar (PDA), minimal medium (MM) or complete medium (CM) for mycelial growth tests, and in mung bean liquid (MBL) medium [40] for sporulation analysis.

### Sequence analysis of *FgVEA*

The *FgVEA* were originally identified through homology searches of the *F. graminearum* genome sequence (available at http://www.broadinstitute.org/annotation/genome/fusarium_group/MultiHome.html) by using BLAST with the *FvVE1* from *F. verticillioides* [13] as query. To verify the existence and the size of intron in *FgVEA*, RNA was extracted from mycelia of the wild-type strain PH-1 using the TaKaRa RNAiso Reagent (TaKaRa Biotech. Co., Dalian, China) and used for reverse transcription with a RevertAid H Minus First Strand cDNA Synthesis kit (Fermentas Life Sciences, Burlington, Canada) according to the manufacturer's instructions. Reverse transcription PCR amplification of *FgVEA* cDNAs were performed using the primer pair Va-F1 + Va-R1 (Table S1). PCR amplifications were performed with the following parameters: initial denaturation at 95°C for 3 min, followed by 35 cycles of denaturation at 94°C for 40 s, annealing at 54°C for 40 s, extension at 72°C for 2 min, and final extension at 72°C for 10 min. The resultant PCR products were purified, cloned and sequenced.

### Construction of vector for the deletion of *FgVEA*

The *FgVEA* gene deletion vector pCA-FgVeA-Del was constructed by inserting two flanking sequences of *FgVEA* gene into two sides of the *HPH* (hygromycin resistance) gene in the pBS-HPH1 vector [41]. The upstream flanking sequence fragment of *FgVEA* was amplified from PH-1 genomic DNA using the primer pair A1 + A2 (Table S1). The 616-bp fragment was inserted into *Xho* I-*Sal* I sites of the pBS-HPH1 vector to generate the plasmid pBS-FgVeA-up. Subsequently, a 554-bp downstream flanking sequence fragment of *FgVEA* was amplified from the PH-1 genomic DNA using the primer pair A3 + A4 and was inserted into the *Hind* III-*BamH* I site of pBS- FgVeA-up vector to generate the plasmid pBS- FgVeA-UD. Finally, the 2,673-bp fragment containing FgVeA-upstream-HPH-FgVeA-downstream cassette (Fig. 1A) was obtained by digestion of plasmid pBS-FgVeA-UD with *Xho* I and *BamH* I, and ligated into the *Xho* I-*Bam*H I site in pCAMBIA 1300 (CAMBIA, Canberra, Australia). The resultant *FgVEA* deletion vector pCA-FgVeA-Del was transformed into *Agrobacterium tumefaciens* strain C_58_C_1_ by electroporation, the *A. tumefaciens*-mediated fungal transformation was performed as described previously [42].

### Complementation of the *FgVEA* gene deletion mutant

The *FgVEA* deletion mutant (ΔFgVeA-9) was complemented with a full-length *FgVEA* gene, to confirm that the phenotype changes in *FgVEA* deletion mutant were due to the deletion of the gene. The *FgVEA* complement plasmid pCA-FgVeA-C was constructed using the backbone of pCAMBIA1300. First, a *Xho* I-*Kpn* I *NEO* cassette containing a *trpC* promoter was amplified from plasmid pBS-RP-Red-A8-NEO [43] with primers neo-F + neo-R (Table S1), and cloned into the *Xho* I-*Kpn* I site of pCAMBIA1300 to create plasmid pCA-neo. Then, a full-length *FgVEA* gene including 2,129-bp promoter region and 854-bp terminator region was amplified from genomic DNA of the wild-type strain PH-1 using the primer pair Va-com-F + Va-com-R (Table S1), and subsequently cloned into *Pst* I and *Hind* III sites of pCA-neo to generate the complement plasmid pCA-FgVeA-C. Before plasmid pCA-FgVeA-C was transformed into *A. tumefaciens* strain C_58_C_1_, *FgVEA* in this plasmid was sequenced to ensure flawlessness of the sequence. Transformation of ΔFgVeA-9 with full-length *FgVEA* gene was conducted as described above except that geneticin was used as a selection agent.

### Mycelial growth and conidiation assays

Mycelial growth tests under different conditions were performed on PDA or MM plates supplemented with the following products: NaCl, KCl, iprodione, fludioxonil, caffeine, congo red, SDS at concentrations indicated in figure legends. Each plate was inoculated with a 5-mm mycelial plug taken from the edge of a 3-day-old colony. There were three replicate plates for each treatment. Plates were incubated at 25°C for 4 days in the dark, and then colony diameter in each plate was measured and the original mycelial plug diameter (5 mm) subtracted from each measurement. The percentage of the mycelial radial growth inhibition (RGI) was calculated using the formula RGI  =  ((C–N)/(C–5))*100, where, C is colony diameter of the control, and N is that of a treatment. Each experiment was repeated three times.

For conidiation assays, ten mycelial plugs (5-mm in diameter) of each strain taken from the periphery of a 3-day-old colony were inoculated in a 50-ml flask containing 10 ml of MBL medium. The flasks were incubated at 25°C for 4 days in a shaker (180 rpm). For each strain, the number of conidia in the broth was determined using a hemacytometer. The experiment was repeated three times.

### Microscopic examination of hyphal and conidial morphology

Hyphal and conidial morphology of the strains were examined with the Leica TCS SP5 imaging system. For transmission electron microscopy, conidia were fixed with 2.5% glutaraldehyde for 24 h at 4°C, then samples were washed with 0.1 M phosphate buffered saline (PBS) for three times, fixed in 1% osmic acid for 3 h at room temperature. After the fixed tissues were rinsed 3 times (15 min each) with 0.1 M PBS, and dehydrated in graded ethanol solutions, the samples were embedded in Lowicryl K4M resin (Electron Microscopy Sciences, Fort Washington, PA, USA). Ultrathin sections (70 nm) were cut from the embedded tissue blocks and mounted onto nickel grids before observation. The sections were examined under an electron microscope JEM-1200EX (JEOL, Japan).

### Histochemical analysis of lipid droplets

Lipid droplets in the conidia were visualized by staining with a Nile Red staining solution [44], [45] consisting of 20 mg/mL polyvinylpyrrolidone and 2.5 µg/mL Nile Red Oxazone (9-diethylamino-5H-benzo[α] phenoxazine-5-one, Sigma) in 50 mM Tris-maleate buffer (pH 7.5). Briefly, after incubation in MBL medium for 3 d, conidia of each strain were harvested and mounted in the Nile Red staining solution. Within a few seconds, lipid droplets began to fluoresce when viewed under a microscope with episcopic fluorescence attachment.

### Determination of intracellular glycerol accumulation

Each strain was grown in potato dextrose broth (PDB) for 2 days at 25°C in a shaker. Mycelia of each strain were harvested and ground in liquid nitrogen. Then, mycelial powder (100 mg) was transferred to a 2-ml microcentrifuge tube containing 0.1 ml glycerol extraction buffer (Shanghai Chaoyan Biotechnology Co.). After mixing by a vortex shaker (HaLiDa, Jiangsu, China) three times for 30 s each, the tubes were centrifuged at 5000 *g* for 20 min. The resulting supernatant was transferred to a new tube, and 10 µl of each supernatant was mixed with 190 µl detection buffer of a glycerol assay kit (Shanghai Chaoyan Biotechnology Co.). After the mixture was incubated at 37°C for 15 min, the glycerol concentration was determined by a spectrophotometer (SPECTRAmax Plus) at 550 nm. The experiment was repeated three times.

### Yeast two hybrid analysis

To construct plasmids for yeast two hybrid screen analysis, the coding sequence of the full length *FgVEA, FgVelB, FgLaeA1, FgVIP1, FgVIP2, FgVIP3, FgVIP4, FgVIP5* or *FgVIP6* was amplified from the cDNA of PH-1. The *FgVelB*, *FgVIP1* and *FgVIP4* fragments were inserted into the *Nde* I-*BamH* I sites of the yeast GAL4 binding domain vector pGBKT7 and GAL4 activation domain vector pGADT7 (Clontech, Mountain View, CA, USA). The *FgVIP3* and *FgVIP5* PCR fragments were inserted into the *Nde* I-*EcoR* I sites of the yeast GAL4 binding domain vector pGBKT7 and GAL4 activation domain vector pGADT7. The *FgLaeA1* and *FgVIP2* PCR fragments were inserted into the *Sma* I-*BamH* I sites of the yeast GAL4 binding domain vector pGBKT7 and GAL4 activation domain vector pGADT7, respectively. The *FgVIP6* fragment was inserted into the *EcoR* I -*BamH* I sites of the yeast GAL4 binding domain vector pGBKT7 and GAL4 activation domain vector pGADT7. The yeast two hybrid plasmids AD-FgLaeA1 + BD-FgVeA, AD-FgVelB + BD-FgVeA, AD-FgVIP1 + BD-FgVeA, AD-FgVIP2 + BD-FgVeA, AD-FgVIP3 + BD-FgVeA, AD-FgVIP4 + BD-FgVeA, AD-FgVIP5 + BD-FgVeA, AD-FgVIP6 + BD-FgVeA were co-transformed into the *S. cerevisiae* reporter strain AH109 according to LiAc/SS-DNA/PEG transformation procedure [46]. The pair of plasmid pGBKT7-53 and pGADT7 was served as a positive control. The pairs of plasmids pGBKT7-Lam and pGADT7, pGADT7 and pGBKT7-FgVeA were used as negative controls. Transformants were grown at 30°C for 72 h on synthetic medium lacking Leu and Trp, and then transferred to the medium lacking His, Leu and Trp and containing 5 mM 3-aminotriazole (3-AT) to identify binding activity. Three independent experiments were performed to confirm yeast two hybrid results.

### SAGE analysis of gene expression profiling in ΔFgVeA-9

The wild type progenitor and ΔFgVeA-9 were grown in PDB for 2 days at 25°C in a shaker. Then mycelia of each strain were harvested and used for RNA extraction. The library constructions used for SAGE analysis were obtained from the total RNA of wild-type strain and ΔFgVeA-9 mutant using the kit for preparing samples for digital gene expression-Tag profiling with DpnII (Illumina Inc., California, USA) according to the manufacturer's protocol. The experiment was performed by BGI Co. (Shenzhen, China) using Illumina Cluster Station and Illumina HiSeq (TM) 2000 System. Since tags detected by SAGE with a frequency less than 3 transcripts per million (tpm) may not be reliable [47], only tags with a frequency ≥3 tpm were used in data analysis in this study. The unique tags were then aligned to all the known transcripts of *F. graminearum* using Novoalign aligner (Novocraft Technologies, Kuala Lumpur, Malaysia). The frequencies of each SAGE tag in the *FgVEA* deletion mutant ΔFgVeA-9 and wild-type strain PH-1 were compared, and the statistical significance (*P* value) was calculated according to Audic and Claverie test using the program IDEG6 [48]. The *P* value is a measure of confidence that the gene is differentially expressed in the two compared samples.

### Pathogenicity assays on flowering wheat heads

After incubation in MBL medium for 4 days, conidia of each strain were collected by filtration through three layers of gauze and subsequently re-suspended in sterile distilled water to a concentration of 1×10^6^ conidia/ml. A10-µl aliquot of conidial suspension was injected into a floret in the central section spikelet of single flowering wheat heads of susceptible cultivar Jimai22. There were ten replicates for each strain. After inoculation, the plants were kept at 22±2°C under 95–100% humidity. Fifteen days after inoculation, the infected spikelets in each inoculated wheat head were recorded. The experiment was repeated for four times.

### Analysis of DON production and expression level of *TRI5* and *TRI6*

A 30-g aliquot of healthy wheat kernels was sterilized and inoculated with 1 ml spore suspension (10^6^ spores/ml) of the wild-type strain PH-1, complemented strain ΔFgVeA-9C and deletion mutant ΔFgVeA-9. After incubation at 25°C for 20 days, DON was extracted using a previously described protocol [49], and the amount of *F. graminearum* DNA in each sample was determined using a quantitative real-time PCR method [50]. The DON extracts were purified with PuriTox^SR^ DON column TC-T200 (Trilogy analytical laboratory), and the amount of DON (per mg fungal DNA) in each sample was determined by using a HPLC system Waters 1525. The experiment was repeated three times independently, and data were analyzed using analysis of variance (SAS version 8.0; SAS Institute, Cary, NC).

To determine expression level of *TIR5* and *TRI6*, the mycelia of the wild-type progenitor PH-1, and the ΔFgVeA-9 were inoculated into GYEP medium (5% glucose, 0.1% yeast extract, 0.1% peptone) and cultured for 2 days at 25°C in the dark. Total RNA was extracted from mycelia of each sample, the expression of *TRI5* and *TRI6* were determined using a quantitative real-time PCR method. The experiment was repeated three times.

## Supporting Information

Figure S1 **Phylogenetic analysis and alignment of VeA proteins from** ***F. graminearum, F. fujikuroi, F. verticillioides, A. nidulans*** **, and** ***P. chrysogenum*** **.** **(A)** Phylogenetic analysis of amino acid sequences of VeA from *F. graminearum, F. fujikuroi, F. verticillioides, A. nidulans* and *P. chrysogenum*. **(B)** Alignment of amino acid sequences of VeA from *F. graminearum* with those from *F. fujikuroi, F. verticillioides, A. nidulans*, and *P. chrysogenum*.
(TIF)

Figure S2 **A total of 1215 up-regulated (red) and 354 down-regulated (green) genes in** ***FgVEA*** **deletion mutant were mapped in chromosomes.**
(TIF)

Figure S3 Effects of *FgVEA* deletion on expression of *F. graminearum* genes involved in peroxisome biogenesis (**A**) and SNARE interactions in vesicular transport pathway (**B**). The up- and down-regulated genes in the *FgVEA* deletion mutant are indicated in red- and green- boxes, respectively. Numbers nearby boxes represent fold changes of gene expression.
(TIF)

Figure S4 **Colony morphology of** ***FgLaeA1,*** **and six** ***FgVIP*** **(FgVeA interacting protein) deletion mutants grown on PDA medium.** The wild-type strain PH-1, *FgLaeA1* deletion mutant ΔFgLaeA1-4, the *FgVIP* deletion mutants ΔFgVIP1-2, ΔFgVIP2-7, ΔFgVIP3-8, ΔFgVIP4-4, ΔFgVIP5-6 and ΔFgVIP6-9 were grown on PDA for 4 days at 25°C. The photos were taken from top **(A)** and bottom **(B)** of plates, respectively.
(TIF)

Figure S5 **The gene expression profiling in** ***FgPTC3*** **and** ***FgVEA*** **deletion mutants.** The 721 genes out of 1,215 up-regulated more than 5 folds in ΔFgVeA-9 were also up-regulated in the *FgPTC3* deletion mutant ΔFgPtc3- 8 (left). The 218 genes out of 354 down-regulated more than 5 folds in ΔFgVeA-9 were also down-regulated in the *FgPTC3* deletion mutant ΔFgPtc3- 8 (right). A total of 1863 up-regulated and 546 down-regulated genes were detected in the two mutants. The expressions of genes were detected by the serial analysis of gene expression method.
(TIF)

Table S1 **Oligonucleotide primers used in this study.**
(DOC)

Table S2 **Expression changes of the genes involved in fatty acid metabolism, cell wall and aurofusarin biosyntheses in** ***F. graminearum FgVEA*** **deletion mutant ΔFgVeA-9 detected by serial analysis of gene expression method.**
(DOC)
