# Supplementary figures and images for: Involvement of a Velvet Protein FgVeA in the Regulation of Asexual Development, Lipid and Secondary Metabolisms and Virulence in Fusarium graminearum
Source: PLoS One. 2011 Nov 29;6(11):e28291. doi: 10.1371/journal.pone.0028291 (PMC3226687; doi:10.1371/journal.pone.0028291)

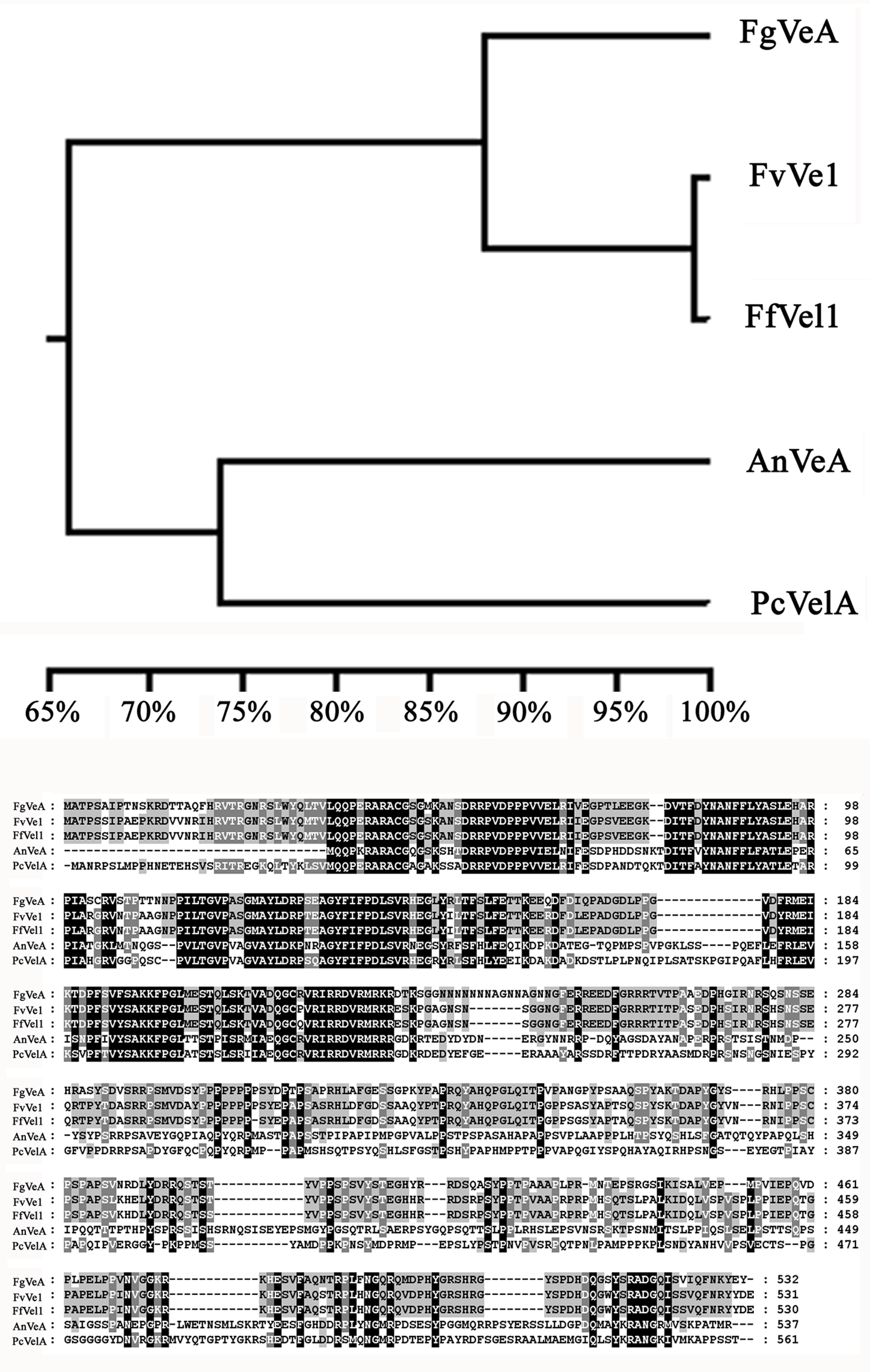

Supplement: Figure S1 — Phylogenetic analysis and alignment of VeA proteins from F. graminearum, F. fujikuroi, F. verticillioides, A. nidulans , and P. chrysogenum . (A) Phylogenetic analysis of amino acid sequences of VeA from F. graminearum, F. fujikuroi, F. verticillioides, A. nidulans and P. chrysogenum. (B) Alignment of amino acid sequences of VeA from F. graminearum with those from F. fujikuroi, F. verticillioides, A. nidulans, and P. chrysogenum. (TIF) [file pone.0028291.s001.tif]

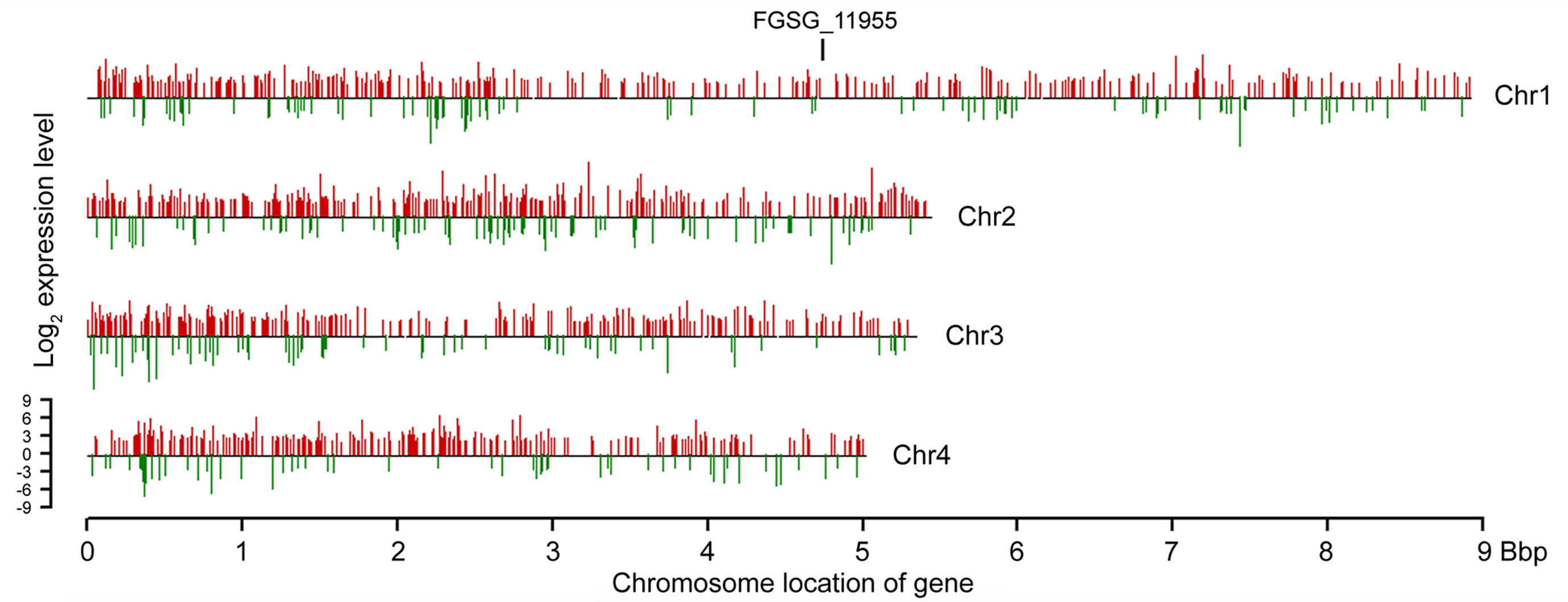

Supplement: Figure S2 — A total of 1215 up-regulated (red) and 354 down-regulated (green) genes in FgVEA deletion mutant were mapped in chromosomes. (TIF) [file pone.0028291.s002.tif]

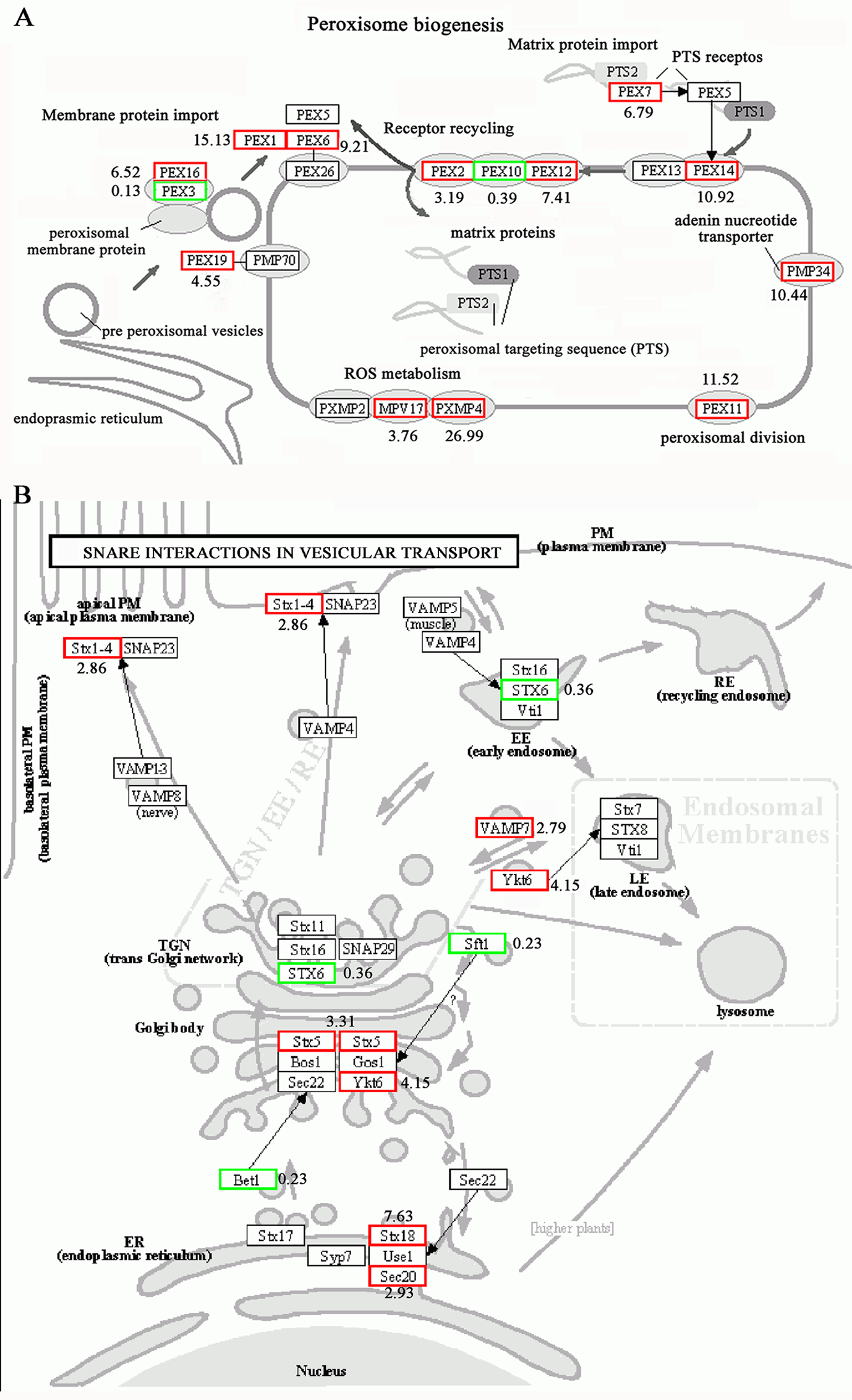

Supplement: Figure S3 — Effects of FgVEA deletion on expression of F. graminearum genes involved in peroxisome biogenesis (A) and SNARE interactions in vesicular transport pathway (B). The up- and down-regulated genes in the FgVEA deletion mutant are indicated in red- and green- boxes, respectively. Numbers nearby boxes represent fold changes of gene expression. (TIF) [file pone.0028291.s003.tif]

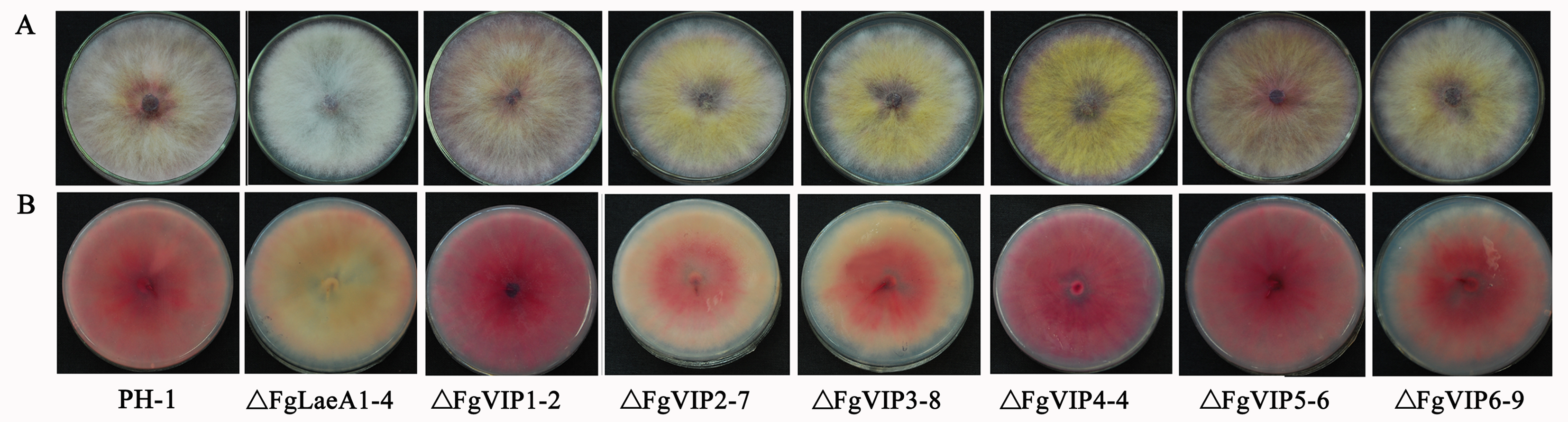

Supplement: Figure S4 — Colony morphology of FgLaeA1, and six FgVIP (FgVeA interacting protein) deletion mutants grown on PDA medium. The wild-type strain PH-1, FgLaeA1 deletion mutant ΔFgLaeA1-4, the FgVIP deletion mutants ΔFgVIP1-2, ΔFgVIP2-7, ΔFgVIP3-8, ΔFgVIP4-4, ΔFgVIP5-6 and ΔFgVIP6-9 were grown on PDA for 4 days at 25°C. The photos were taken from top (A) and bottom (B) of plates, respectively. (TIF) [file pone.0028291.s004.tif]

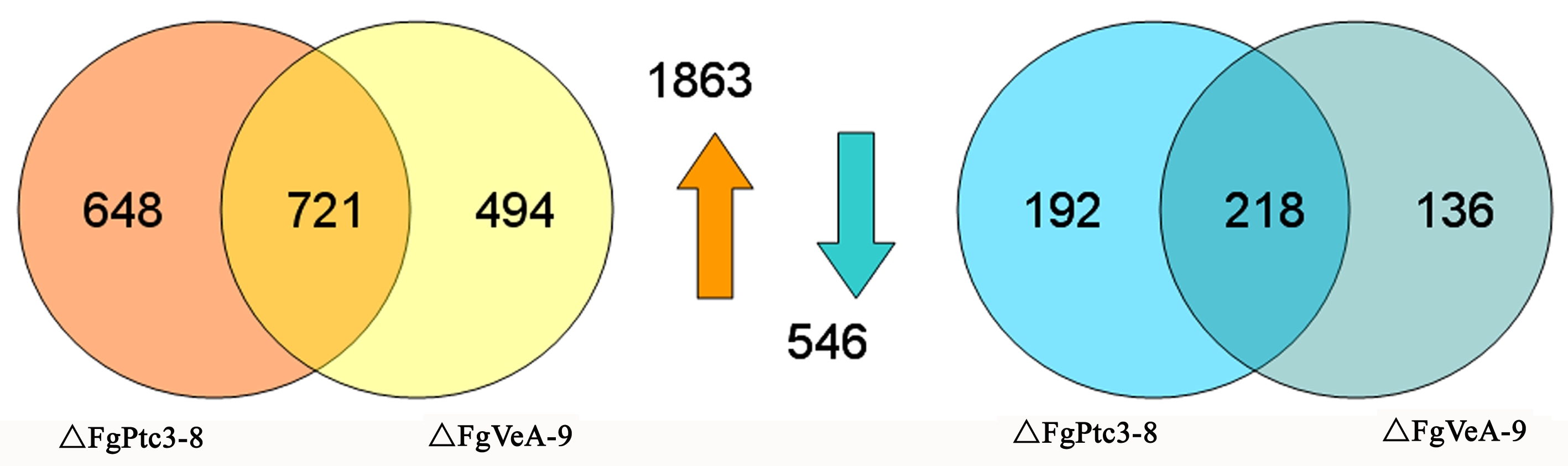

Supplement: Figure S5 — The gene expression profiling in FgPTC3 and FgVEA deletion mutants. The 721 genes out of 1,215 up-regulated more than 5 folds in ΔFgVeA-9 were also up-regulated in the FgPTC3 deletion mutant ΔFgPtc3- 8 (left). The 218 genes out of 354 down-regulated more than 5 folds in ΔFgVeA-9 were also down-regulated in the FgPTC3 deletion mutant ΔFgPtc3- 8 (right). A total of 1863 up-regulated and 546 down-regulated genes were detected in the two mutants. The expressions of genes were detected by the serial analysis of gene expression method. (TIF) [file pone.0028291.s005.tif]
