# Supplementary material for: Involvement of a Velvet Protein FgVeA in the Regulation of Asexual Development, Lipid and Secondary Metabolisms and Virulence in Fusarium graminearum
Source: PLoS One. 2011 Nov 29;6(11):e28291. doi: 10.1371/journal.pone.0028291 (PMC3226687; doi:10.1371/journal.pone.0028291)
Supplement: Table S1 — Oligonucleotide primers used in this study. (DOC) [file pone.0028291.s006.doc]

**Table S1 Oligonucleotide primers used in this study**

| Primer code | Primer | Sequence (5’-3’)^a^ | Relevant characteristics |
| --- | --- | --- | --- |
| 1 | Va-F1 | catatgATGGCGACACCTTCAGC | A pair of PCR primers for amplification of the full cDNA sequence of the *FgVEA* gene |
| 2 | Va-R1 | ggatccTTAATACTCGTATTTGTTG |
|  |  |  |  |
| 3 | A1 | ATctcgagCCTCCGTCCGTTCTTGAATTA | A pair of PCR primers for amplification of the 616-bp *FgVEA* upstream fragment for construction of the gene deletion vector |
| 4 | A2 | ATgtcgacGCATAGAGAAAGAAGTTGGCG |
|  |  |  |  |
| 5 | A3 | ATaagcttTGGTCAGATCAGCGTTATTCA | A pair of PCR primers for amplification of the 554-bp *FgVEA* downstream fragment for construction of the gene deletion vector |
| 6 | A4 | ATggatccCATGGCTTTACCCTTGATCCT |
|  |  |  |  |
| 7 | A5 | TTGACCCTCCGCCTGTTGT | A pair of PCR primers for identification of *FgVEA* deletion mutants |
| 8 | A6 | TTGTCGCTCATGTATCTTCCA |
|  |  |  |  |
| 9 | A-probe-F | GAACAAGAACAACAACGGCA | PCR primers to amplify the 1,162-bp *FgVEA* fragment used as the probe for Southern blot analysis |
| 10 | A-probe-F | GCATAGAGAAAGAAGTTGGCG |
|  |  |  |  |
| 11 | Va-com-F | ATctgcagTCTTCGCTTAAACTGGGCTG | A pair of PCR primers to amplify the full *FgVEA* including 2,129-bp up- and 854-bp down-fragments |
| 12 | Va-com-R | ATaagcttAATTTCCATCAAGCTGGCCT |
|  |  |  |  |
| 13 | FgVelB-F | catatgATGGCGGTGGTGGGAACCC | PCR primers for amplification of full cDNA sequence of the *FgVelB* gene |
| 14 | FgVelB-R | ggatccTCAGTTCTGATCGTACATC |
|  |  |  |  |
| 15 | FgLaeA1-F | cccgggTATGGCTGTGATGCCTCCA | PCR primers for amplification of full cDNA sequence of the *FgLaeA1* gene |
| 16 | FgLaeA1-R | ggatccTCAGATGGTGCTTATGAGCTTC |
|  |  |  |  |
| 17 | FgV1-F | catatgATGGCTGTCGTTGAAGCAGAT | PCR primers for amplification of the full cDNA sequence of the *FgVIP1* gene |
| 18 | FgV1-R | ggatccCTACGCGTCAGAGCTCTCA |
|  |  |  |  |
| 19 | FgV2-F | cccgggTATGGCTTCTGAGCAGGAAACT | PCR primers for amplification of the full cDNA sequence of the *FgVIP2* gene |
| 20 | FgV2-R | ggatccCTACGCTTGAAGTGGCTTTCG |
|  |  |  |  |
| 21 | FgV3-F | catatgATGGGTGGTCAAGAATCAGGA | PCR primers for amplification of the full cDNA sequence of the *FgVIP3* gene |
| 22 | FgV3-R | gaattcTTACCCATCCAATGGCTTCTG |
|  |  |  |  |
| 23 | FgV4-F | catatgATGTCTTCTCCTCGCAACAA | PCR primers for amplification of the full cDNA sequence of the *FgVIP4* gene |
| 24 | FgV4-R | ggatccTCACTCGACAGCCTGTGCCTT |
|  |  |  |  |
| 25 | FgV5-F | catatgATGGACCAGGAACCACGCAA | PCR primers for amplification of the full cDNA sequence of the *FgVIP5* gene |
| 26 | FgV5-R | gaattcTTAGTACGGCTTCTGCCCATA |
|  |  |  |  |
| 27 | FgV6-F | gaattcATGGCCGACAACACTGCTT | PCR primers for amplification of the full cDNA sequence of the *FgVIP6* gene |
| 28 | FgV6-R | ggatccCTACTCAGGCTTCCTCCCATA |
|  |  |  |  |
| 29 | Os2-F | TTGTCAAGTCGCTACCCAAG | PCR primers for amplification of the partial *FgOS2* gene in quantitative real-time PCR assays |
| 30 | Os2-R | ATGTATCAACAGGCAGATCGG |
|  |  |  |  |
| 31 | Gls2-F | TCTGCCGATTTCGTTCTTG | PCR primers for amplification of the partial *FgGLS2* gene in quantitative real-time PCR assays |
| 32 | Gls2-R | ACCCTGCATAGGAACCATCTT |
|  |  |  |  |
| 33 | Tri5-F | TCACCCAGGAAACCCTACACT | PCR primers for amplification of the partial *TRI5* gene in quantitative real-time PCR assays |
| 34 | Tri5-R | ACGTTTGCCAGTTGTGCAA |
|  |  |  |  |
| 35 | Tri6-F | ATGATTTACATGGAGGACGA | PCR primers for amplification of the partial *TRI6* gene in quantitative real-time PCR assays |
| 36 | Tri6-R | TCAACCCTTGTGTATCCGC |
|  |  |  |  |
| 37 | Mkk1-F | GGATTCAACAAAGAGTGCGCT | PCR primers for amplification of the partial *FgMKK1* gene in quantitative real-time PCR assays |
| 38 | Mkk1-R | ACCGAAATCGCAAAGCTTGA |
|  |  |  |  |
| 39 | Slt2-F | TTCTTCACATTCTCGGAACCC | PCR primers for amplification of the partial *FgSLT2* gene in quantitative real-time PCR assays |
| 40 | Slt2-R | CATCCAAAATCATACCACGCA |
|  |  |  |  |
| 41 | Pks12-F | AATGGCTTCTTGCACATTTCC | PCR primers for amplification of the partial *PKS12* gene in quantitative real-time PCR assays |
| 42 | Pks12-R | GCAATCCGATCCATGAACAA |
|  |  |  |  |
| 43 | AurJ-F | AAAAAGCAGCCAAGGAGCAT | PCR primers for amplification of the partial *AURJ* gene in quantitative real-time PCR assays |
| 44 | AurJ-R | TTCTGATGACACGCTCCCGTA |
|  |  |  |  |
| 45 | hph-F | GACGTTGTAAAACGACGGCC | PCR primers for amplification of hygromycin resistance gene (*hph*) |
| 46 | hph-R | AATTCGTCGACGTTAACTGGCTG |
|  |  |  |  |
| 47 | neo-F | ATctcgagGGAGGTCAACACATCAATGCT | PCR primers for amplification of neomycin resistance gene (*neo*) |
| 48 | neo-R | ATggtaccTCAGAAGAACTCGTCAAGAAG |
|  |  |  |  |
| 49 | actin-F | ATCCACGTCACCACTTTCAA | PCR primers for amplification of the reference actin gene in quantitative real-time PCR assays |
| 50 | actin-R: | TGCTTGGAGATCCACATTTG |
|  |  |  |  |

^a^ The respective restriction enzyme sites included in primers are listed in lowercase in the sequence.
