# Supplementary material for: Involvement of a Velvet Protein FgVeA in the Regulation of Asexual Development, Lipid and Secondary Metabolisms and Virulence in Fusarium graminearum
Source: PLoS One. 2011 Nov 29;6(11):e28291. doi: 10.1371/journal.pone.0028291 (PMC3226687; doi:10.1371/journal.pone.0028291)
Supplement: Table S2 — Expression changes of the genes involved in fatty acid metabolism, cell wall and aurofusarin biosyntheses in F. graminearum FgVEA deletion mutant ΔFgVeA-9 detected by serial analysis of gene expression method. (DOC) [file pone.0028291.s007.doc]

**Table S2 Expression changes of the genes involved in fatty acid metabolism, cell wall and aurofusarin biosyntheses in *F. graminearum* *FgVEA* deletion mutant ΔFgVeA-9 detected by serial analysis of gene expression method.**

| Pathway | Accession number | Putative function | Fold change in gene expression^a^ |
| --- | --- | --- | --- |
| Fatty acid biosynthesis | FGSG_05322 | fatty acid synthase subunit beta dehydratase | 0.53 |
|  | FGSG_05321 | fatty acid synthase subunit alpha reductase | 0.32 |
|  | FGSG_07226 | 3-oxoacyl-[acyl-carrier-protein] synthase, mitochondrial precursor | 1.68 |
|  | FGSG_02324 | hypothetical protein similar to type I polyketide synthase | 1.83 |
|  | FGSG_02210 | conserved hypothetical protein | 22.4 |
|  | FGSG_07223 | hypothetical protein similar to short chain dehydrogenase family protein | 41.34 |
|  | FGSG_03838 | conserved hypothetical protein | 1.59 |
|  | FGSG_03375 | conserved hypothetical protein (953 nt);Pfam:PF00106.17 | 1.79 |
|  | FGSG_11409 | conserved hypothetical protein | 12.93 |
|  | FGSG_01857 | hypothetical protein similar to 3-oxoacyl-acyl-carrier-protein reductase | 20.93 |
|  | FGSG_10026 | hypothetical protein similar to 3-oxoacyl-(acyl-carrier-protein) reductase | 3.55 |
|  | FGSG_08816 | conserved hypothetical protein | 2.6 |
| Fatty acid metabolism | FGSG_07833 | conserved hypothetical protein | 21.53 |
|  | FGSG_01419 | hypothetical protein similar to AMP-binding protein | 25.48 |
|  | FGSG_07277 | conserved hypothetical protein | 21.76 |
|  | FGSG_08843 | hypothetical protein similar to AMP dependent CoA ligase | 42.43 |
|  | FGSG_13860 | conserved hypothetical protein | 4.65 |
|  | FGSG_09424 | hypothetical protein similar to fadD35 | 55.89 |
|  | FGSG_07659 | hypothetical protein similar to fadD36 | 6.57 |
|  | FGSG_01415 | conserved hypothetical protein | 2.38 |
|  | FGSG_08543 | hypothetical protein similar to Fum16p | 2.75 |
|  | FGSG_03363 | hypothetical protein similar to Fum16p | 0.16 |
|  | FGSG_02287 | hypothetical protein similar to acyl-CoA oxidase | 40.83 |
|  | FGSG_05140 | hypothetical protein similar to acyl-CoA dehydrogenase family protein | 40.21 |
|  | FGSG_02379 | conserved hypothetical protein | 2.86 |
|  | FGSG_12573 | hypothetical protein similar to enoyl-CoA hydratase/isomerase family protein | 33.96 |
|  | FGSG_05551 | hypothetical protein similar to peroxisomal D3,D2-enoyl-CoA isomerase | 55.62 |
|  | FGSG_09979 | hypothetical protein similar to enoyl-CoA hydratase/isomerase family protein | 45.13 |
|  | FGSG_12529 | predicted protein | 1.98 |
|  | FGSG_13880 | conserved hypothetical protein | 5.07 |
|  | FGSG_13111 | enoyl-CoA hydratase, mitochondrial precursor | 7.24 |
|  | FGSG_03244 | hypothetical protein similar to enoyl-CoA hydratase/isomerase family protein | 0.31 |
|  | FGSG_07019 | hypothetical protein similar to mitochondrial 3-hydroxyisobutyryl-CoA hydrolase | 9.14 |
|  | FGSG_03546 | hypothetical protein similar to dehydrogenase | 37.94 |
|  | FGSG_00809 | 3-hydroxyacyl-CoA dehydrogenase type-2 | 6.95 |
|  | FGSG_09503 | 3-ketoacyl-CoA thiolase | 64.46 |
|  | FGSG_04243 | 3-ketoacyl-CoA thiolase | 34.17 |
|  | FGSG_13398 | 3-ketoacyl-CoA thiolase B | 5.21 |
|  | FGSG_01581 | conserved hypothetical protein | 13.37 |
|  | FGSG_05087 | acetyl-CoA acetyltransferase | 8.35 |
|  | FGSG_09321 | acetyl-CoA acetyltransferase IB | 0.51 |
| Aurofusarin biosynthesis | FGSG_02324 | hypothetical protein similar to type I polyketide synthase | 0.02 |
|  | FGSG_02328 | hypothetical protein similar to brown 2 (dimerize two 9-hydroxyrubrofusarin) | 0.03 |
|  | FGSG_02320 | hypothetical protein similar to C6 zinc finger protein  (Positive acting transcription factor for the aurofusarin gene cluster) | 0.14 |
|  | FGSG_02326 | conserved hypothetical protein (O-methyltransferase: converts nor-rubrofusarin into rubrofusarin) | 0.02 |
|  | FGSG_02327 | conserved hypothetical protein (Monooxygenase: converts rubrofusarin into 9-hydroxyrubrofusarin) | 0.02 |
|  | FGSG_02321 | conserved hypothetical protein (catalyses the oxidation of dimeric) | 0.03 |
|  | FGSG_02323 | hypothetical protein similar to AurR2 | 0.07 |
| Cell wall boisynthesis | FGSG_07946 | 1,3-beta-glucan synthase component GLS2 | 2.77 |

^a^ Fold-change value represents the fold expression in *FgVEA* deletion mutant ΔFgVeA-9 as compared with that in the wild-type strain PH-1.
